# GAD65 Antibody‐Associated Neurologic Syndrome Overlapping Hemophagocytic Lymphohistiocytosis

**DOI:** 10.1111/cns.70364

**Published:** 2025-03-28

**Authors:** Ya Chen, Doujia Chen, Zhongxiang Xu, Zucai Xu

**Affiliations:** ^1^ Department of Neurology Affiliated Hospital of Zunyi Medical University Zunyi China; ^2^ Department of Hematology The Second Affiliated Hospital of Zunyi Medical University Zunyi China; ^3^ Key Laboratory of Brain Function and Brain Disease Prevention and Treatment of Guizhou Province Zunyi China

**Keywords:** antibody, GAD65, hemophagocytic lymphohistiocytosis, neurologic syndrome

1

Glutamic acid decarboxylase 65 (GAD65) antibody‐associated neurologic syndrome is a rare neurologic syndrome mediated by autoimmune response injury including autoimmune epilepsy, autoimmune cerebellar ataxia, stiff‐person syndrome, and limbic encephalitis [1]. Hemophagocytic lymphohistiocytosis (HLH) is a systemic inflammatory disease that can present with a variety of clinical manifestations [2]. Both are associated with immunoinflammation, but no coexistence has been reported. Here, we report the first case of GAD65 antibody‐associated neurologic syndrome overlap hemophagocytic lymphohistiocytosis.

A previously healthy 49‐year‐old man had a first generalized tonic‐clonic seizure in May 2022. Two months after the initial presentation, he presented symptoms associated with cerebellar dysfunction, including dizziness, unstable walking. In September, he had another seizure with electroencephalogram showing low‐to‐medium amplitude sharp waves scattered in the focal left hemisphere and was treated with sodium valproate sustained‐release tablets 500 mg twice daily. He presented with progressive dizziness and instability. In January 2023, he showed left tinnitus, deafness, diplopia, and was admitted to our hospital.

The neurologic examination revealed slight bradylalia, horizontal gaze nystagmus, diplopia but no limitation of eye movement, diminished tingling on the left side of the face, and an unstable heel‐to‐shin test. Brain magnetic resonance imaging (MRI) showed multiple abnormal signals without contrast enhancement lesions (Figure 1A). Electroencephalogram showed normal. An extensive screening for rheumatological disorders showed negative results, as did screening tests for metabolic, tumor, and infectious causes. Cerebrospinal fluid (CSF) examination revealed normal open pressure, 10 × 10^6^/L leukocytes, 597 mg/L total protein, no heteromorphic cells, and normal glucose and chloride levels. There were CSF‐specific oligoclonal bands (type II). Mitochondrial genetic testing, broad CSF microbiological examination, and CSF metagenomic next‐generation sequencing were unremarkable. Other autoimmune encephalitis and paraneoplastic antibodies remained negative except positive anti‐GAD65 antibodies detected by cell‐based immunoassays in serum (1:30) and in CSF (1:10). After excluding other diseases, he was diagnosed with GAD65 antibody‐associated neurologic syndrome. Meanwhile, hidden‐malignancy screenings for chest, abdomen computed tomography, thyroid, superficial lymph nodes and breast ultrasound, gastroscopy, and colonoscopy were negative. He was prescribed intravenous methylprednisolone pulse therapy (1 g/day) for 5 days and gradually reduced the dosage to oral tapering prednisone, which alleviated his symptoms, especially diplopia and dizziness. The patient had been seizure‐free since taking valproate.

**FIGURE 1:**
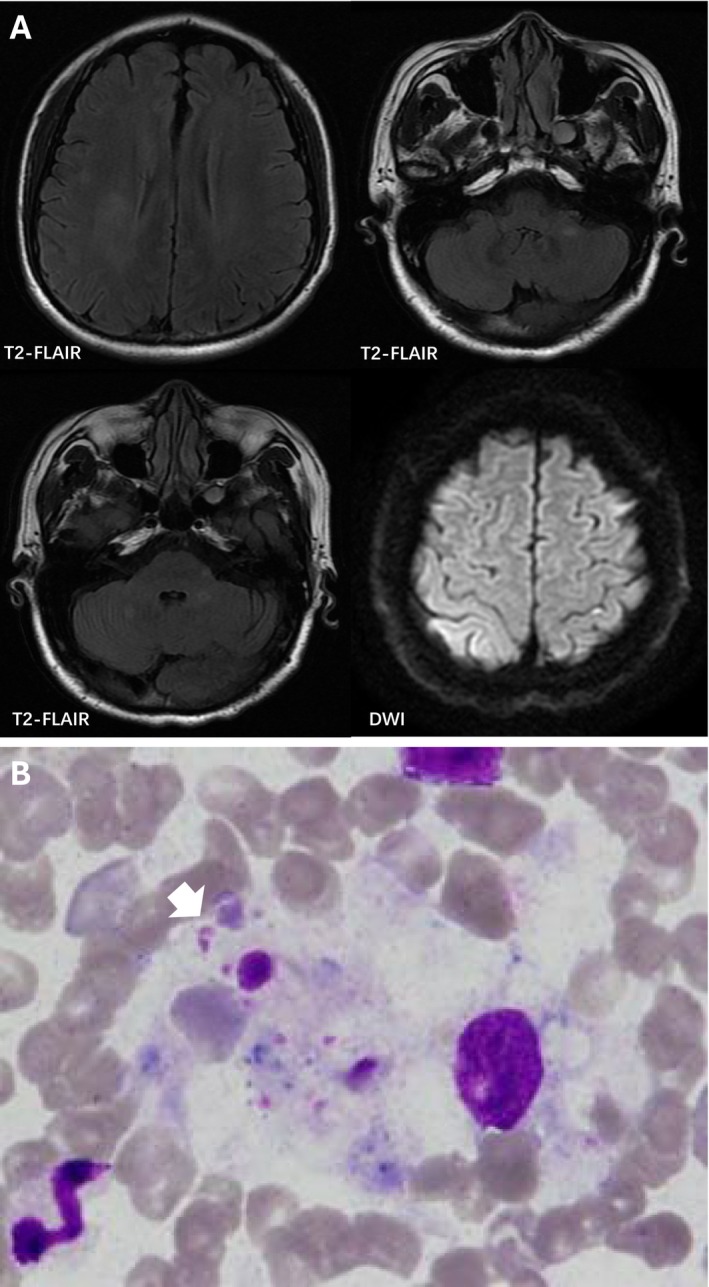
The imaging and bone marrow smear findings of the patient. (A) T2‐weighted fluid‐attenuated inversion recovery (T2‐FLAIR) sequences showed analogous round, plaque‐like, ill‐defined demyelination in bilateral frontoparietal white matter, cerebellar regions and left pontine arm and with absence of contrast enhancement. Diffusion weighted imaging (DWI) revealed cortical hyperintense signals. (B) Bone marrow cytology showed histiocytic phagocytosis (white arrow) with engulfed erythrocytes, platelets, and cellular debris (Wright–Giemsa staining, ×1000).

After being discharged for 30 days, the patient became very weak and was readmitted for abdominal distension and pain. A routine blood examination showed a significant decrease in white blood cells and platelets and significant increases in fibrinogen, ferritin, triglycerides, D‐dimer, hypohepatia, and myocardial enzyme spectrum. Related examinations for infection were negative. Abdominal CT only indicated splenomegaly. The bone marrow cytology noted histocytes phagocytosing hemocytes (Figure 1B). The patient was in critical condition with multi‐organ dysfunction and died. The family declined an autopsy.

The patient showed rare overlap with epilepsy and cerebellar ataxia, which are main features in GAD65 antibody‐associated neurologic syndrome [3]. He underwent extensive examinations to exclude other potential diagnoses. The diagnosis of GAD65 antibody‐associated neurologic syndrome is based on clinical manifestations, the detection of anti‐GAD65 antibodies in both serum and CSF [4]. Therefore, he was diagnosed with GAD65 antibody‐associated neurologic syndrome.

The pathophysiologic mechanisms of anti‐GAD65 in neuroinflammation are still unclear. It is challenging to identify whether there is a direct antibody‐associated pathogenic role because GAD65 is located intracellularly not accessible for extracellular antibodies. it has been hypothesized that GAD65 might transiently appear on the cell surface in the synaptic cleft during the process of neurotransmission and exocytosis [5]. Histologically, neuronal loss and infiltrating T cells are seen in patients with anti‐GAD65 antibody‐associated neurologic syndromes [6]. This supports the idea that immune reactions are relevant in these patients. In the pathologic study of GAD65 antibody‐related encephalitis, a cytotoxic T‐cell response is the main pathologic mechanism [7].

The patient fulfilled HLH‐2004 diagnostic criteria: splenomegaly, bicytopenia, hypertriglyceridemia, hypofibrinogenemia, histiocytic hemophagocytosis in marrow, and hyperferritinemia [2]. Adult HLH has a complicated etiology including infection, autoimmune disease, or malignancy [8]. However, no tumor or infection evidence was found in this patient. Therefore, we hypothesize that immunoinflammatory imbalances, mediated by anti‐GAD65 antibodies, significantly contribute to the development of HLH. HLH is a hyperinflammatory syndrome characterized by overactivation of lymphocytes and macrophages in association with high levels of cytokines [9]. There are some similar immune mechanisms between anti‐GAD65 antibody‐associated neurologic syndrome and HLH. We speculate that GAD65 antibodies trigger cytotoxic T lymphocyte‐mediated autoimmune response, and unchecked cytotoxic T cells may lead to overactivation of the immune system, disrupting immune surveillance and host defense systems, leading to dramatic increase of pro‐inflammatory cytokines. This cytokine storm drives the activation of macrophages, potentially exacerbating tissue damage and inflammation.

Some limitations are as follows. Firstly, the etiology of brain lesions remains histologically unconfirmed due to the family's declination of postmortem. Second, while surveillance evaluations during the survival period demonstrated no evidence of malignancy, GAD65‐IgG positivity overlapping HLH raised clinical concern for underlying occult neoplasia due to established paraneoplastic associations between the two diseases and malignancy [7, 10]. Regrettably, the confirmation of malignancy through longitudinal follow‐up was precluded by the patient's demise, leaving the paraneoplastic correlation undetermined in this case.

Our case describes a hitherto undefined phenotype that the co‐occurrence of anti‐GAD65 antibody and HLH in the patient. Our finding shows the underlying pathophysiological relationship and poor prognosis.

## 2 Author Contributions

Y.C., D.J.C., Z.X.X., and Z.C.X.: Study design or data acquisition or data analysis/interpretation. Y.C. and Z.C.X.: Manuscript drafting or manuscript revision. All authors have agreed both to be personally accountable for the author's contributions and to ensure that questions related to the accuracy or integrity of any part of the work.

## 3 Ethics Statement

The case report was approved and supervised by the Ethics Committee of the Affiliated Hospital of Zunyi Medical University (No. KLL‐2024‐076). Informed consent was obtained from the patient's family.

## 4 Conflicts of Interest

The authors declare no conflicts of interest.

## Data Availability

Data sharing is not applicable to this article as no new data were created or analyzed in this study.
